# The reciprocal relationship between maternal infant-directed singing and infant gaze

**DOI:** 10.1177/10298649251385676

**Published:** 2025-12-16

**Authors:** Susanne Reisner, Trinh Nguyen, Pierre Labendzki, Stefanie Hoehl, Gabriela Markova

**Affiliations:** 1 Department of Developmental and Educational Psychology, Faculty of Psychology, University of Vienna, Austria; 2 Vienna Doctoral School Cognition, Behavior and Neuroscience, University of Vienna, Austria; 3 Neuroscience of Perception and Action Lab, Italian Institute of Technology, Italy; 4 Department of Developmental and Biological Psychology, Heidelberg University, Germany; 5 Department of Psychology, University of East London, UK; 6 Institute for Early Life Care, Paracelsus Medical University, Austria

**Keywords:** infant-directed singing, spectral flux, acoustic variability, infant attention, infant–caregiver interaction, cross-modal reciprocity

## Abstract

Infant-directed (ID) playsongs and lullabies have distinct acoustic properties connected to their functions to elicit and diffuse infant attention, respectively. In the performative context of ID singing, it is crucial that infants and caregivers adjust to each other for the songs’ function to be reached. In this study, we observed face-to-face ID singing between mothers and their 7-month-old infants and measured variability in maternal singing (i.e., spectral flux) around the onset of infant social gaze toward the mother. Results showed that maternal acoustic variability and infant attention were increased in playsongs over lullabies. Furthermore, mothers increased their acoustic variability both before and after the onset of infant social gaze, especially in playsongs. These findings suggest that mothers increase acoustic variability both to modulate and respond to infant attention, and infants respond to more variable singing by paying more attention to the singing caregiver. Thus, we propose that ID singing interactions are reciprocal, linking infant attentional displays and maternal acoustic responses.

Infant-directed (ID) singing occurs in cultures all over the world (Mehr et al., 2019; Mehr & Krasnow, 2017; Trehub, 2019; Trehub et al., 1993), and caregivers routinely engage in ID singing with their infants (Steinberg et al., 2021; Trehub et al., 1997). A growing number of studies have investigated the acoustic qualities of ID singing and infant reactions to it separately. Yet, it is likely that there is a reciprocal relationship between fine-grained acoustic changes in maternal singing and infant attention to achieve effective communication through different modalities. Here, we investigate the reciprocity between infant attention and maternal ID singing in a face-to-face interaction.

ID singing is especially attention-grabbing for infants (for a review, see Nguyen, Flaten, et al., 2023), even more so than ID or adult-directed speech (Fernald et al., 1989; Nakata & Trehub, 2004; Outters et al., 2020; Tsang et al., 2017). Infants look longer at mothers during ID singing than during speech (Nakata & Trehub, 2004), and they pay above-chance levels of attention to caregivers’ eyes around the beat of ID singing (Lense et al., 2022). Likewise, caregivers make ID singing engaging by modifying their facial expressions and eye movements (Español & Shifres, 2015) to highlight salient information to the beat of their singing (Lense et al., 2022). Moreover, playful singing was found to be positively associated with dyadic gaze coordination during early face-to-face interactions (Markova et al., 2020). Thus, infant–caregiver musical interactions exhibit an intricate interplay of attentional displays and mutual adjustment through multiple modalities.

Caregivers sing to their infants in different caretaking contexts. Depending on their communicative intent, they choose between two broad categories of ID songs: Lullabies to soothe infants and divert attention, and playsongs to engage and direct infants’ attention (Cirelli et al., 2020; Rock et al., 1999; Trehub, 2018; Trehub & Trainor, 1998). These functionalities are linked to the ID songs’ acoustic qualities. Playsongs are usually sung faster, higher and more variable in pitch, and louder than lullabies (e.g., Cirelli et al., 2020; Rock et al., 1999; Trehub & Trainor, 1998).

For both song types, caregivers and infants need to reciprocally adjust to one another for the songs’ communicative function to be reached. In early social interactions, caregivers and infants coordinate their actions to engage and influence each other’s behaviour (Caulfield, 1995; Jaffe et al., 1973). Infants direct caregiver attention through a variety of vocal, affect, and gestural displays and, thus, play an active role in their social interactions from birth. Caregivers respond age-appropriately to infant displays (Caulfield, 1995). In the more performative context of ID singing, caregivers may vary their songs over time to achieve this adjustment (Nguyen, Reisner, et al., 2023). These variations can be captured by time-variant methods, such as spectral flux. Spectral flux measures both frequency and amplitude changes over time (Müller, 2015). Unlike methods focusing solely on amplitude changes, spectral flux thus captures spectro-temporal information in the audio stream (Weineck et al., 2022). This allows for detecting changes, such as pitch variations, that might be missed by examining the amplitude envelope alone. As a result, spectral flux provides a more accurate and holistic representation of musical events since frequency and amplitude modulations can occur independently (Zeng et al., 2005). Interestingly, a previous study found that caregivers adjust the level of spectral flux contained in ID speech to their preterm infant’s wakefulness state (Saliba et al., 2020). These findings suggest that increased spectral flux may prompt infants to interact with caregivers, and in contrast, lower spectral flux may help avoid overstimulating infants and create a calmer environment.

In this study, we examined the reciprocity between live maternal ID singing and 7-month-old infants’ attention. More specifically, we assessed the spectral flux of maternal ID singing of a playsong and a lullaby during a semi-naturalistic musical interaction around the onsets of infant social gaze toward the mother. We aimed to test whether changes in ID singing drive changes in infant attention, changes in infant attention prompt changes in maternal singing, or changes in maternal singing and infant attention co-occur. Accordingly, we hypothesized that maternal singing would increase in variability (i.e., show increased spectral flux) around times of increased infant attention (i.e., around the onset of infant social gaze toward their mother), either as a tool to grab infant attention (if occurring before infant social gaze onset) or as a reaction to infant attention (if occurring after infant social gaze onset). We also hypothesized that this interplay of infant and caregiver adjustment is especially important in playsongs because of their proposed function to attract attention (Cirelli et al., 2020). Therefore, we expected playsongs to contain higher spectral flux than lullabies and to induce more instances of infant social gaze.

## Methods

### Participants

We included 74 7-month-old infants (33 girls; age: 227.54 ± 6.96 days [*M* ± *SD*]) and their mothers (age: 34.58 ± 4.53 years) in the final sample. We decided on this age group because 6 and 7-month-old infants are especially attracted to ID singing (Tsang et al., 2017). We excluded 29 additional mother–infant dyads from the final analysis due to infant fussiness (*n* = 12), equipment failure (*n* = 5), no infant social gaze occurrence in both singing conditions (*n* = 2), maternal failure to follow instructions (*n* = 5), incomplete experiment (*n* = 4), and excessive background noise (*n* = 1).

We recruited participants from a database of families who had expressed interest in and consented to be contacted about partaking in developmental research. These families were recruited in neonatal units at local hospitals, in mother–child activity classes, and through social media. All infants were born full-term (gestational age of 36–42 weeks), had a birth weight of >2,500 g, and had no known developmental delays or neurological or hearing impairments. Infants grew up in predominantly German-speaking households. Mothers were highly educated, with 87.8% holding a university degree. Overall, 70.3% of mothers were first-time mothers and had no other children, 17.6% had one older child, one mother had a same-aged twin, one mother had three older children, and 9.3% provided no information on birth order. The primary caregiver was the mother in 68.9% of participants, both mother and father in 13.5% of participants, the mother and sibling in one participant, and 16.2% provided no information on the primary caregiver. 97.3% of mothers reported playing and singing at least one hour every week in the presence of their infant (*M* = 11.7 ± 11 h listened to, *M* = 6.7 ± 5.9 h sung). 56.8% of mothers reported playing an instrument, and 20.3% sang in a choir or a band. The study was conducted according to the Declaration of Helsinki, approved by the University of Vienna’s ethics committee, and caregivers provided written informed consent.

### Procedure

**Figure 1.**
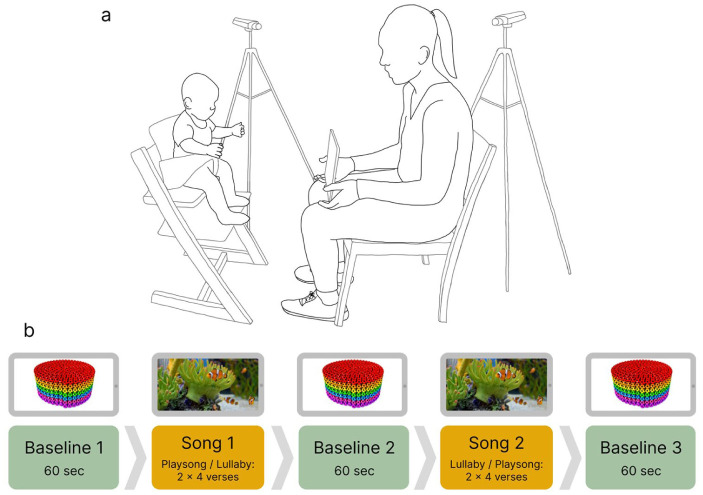
(a) Experimental setup illustration. The infant sat facing their mother in a highchair or a car seat held by an experimenter (not depicted). (b) Procedure. Mothers sang two repetitions of four verses for each lullaby and playsong. During both conditions, a tablet held by the mother played a video of fish swimming in an aquarium. A 60 s baseline, during which the mother did not interact with the infant, preceded and followed each song, during which the tablet showed moving geometric shapes without sound.

During the experiment, infants sat in a car seat or highchair while their mothers faced them, holding a tablet (Figure 1[a]). Infants were seated in a car seat or highchair, depending on electroencephalogram (EEG) or infant motion measurements detailed in another paper (Nguyen, Reisner, et al., 2023), or according to their preference. Each dyad was observed during two experimental singing blocks (Figure 1[b]), the order of which was randomized between participants. A 60 s baseline preceded and followed each singing block (i.e., three baseline blocks in total), during which infants and mothers watched silent videos of slowly moving shapes. We included this break to give dyads some rest between the two singing conditions. Mothers were asked not to talk during baseline blocks but to reciprocate their infants’ communicative attempts via facial expressions and gestures (e.g., smiling, pointing at the tablet). Per singing block, mothers were instructed to sing either a playsong or a lullaby. We selected well-known songs per category, “Schlaf Kindlein, schlaf” (lullaby) and “Es tanzt ein Bibabutzemann” (playsong) (see Figure 1S in the Supplemental material). While mothers were given song recordings to prepare before the experiment, most of them were familiar with the playsong (91%) and the lullaby (96%). On average, mothers sang both the playsong (*M* = 2.48, on a Likert scale from 1 = *never* to 5 = *very often*) and the lullaby (*M* = 2.77 on the Likert scale) moderately often to their infants at home. To ensure that mothers differentiated between the two songs, they were prompted with a metronome before the start of each singing block (i.e., playsong = 170 bpm; lullaby = 100 bpm). The metronome was stopped before the mothers began to sing. Mothers were asked to sing to their infants as they naturally would, and thus they did not have to follow the particular score for each song. All mothers sang two repetitions of four verses of each song. During the singing conditions, the tablet played a calm aquarium video to help with infant fussiness. Three cameras (synchronized in VideoSyncPro, Mangold International) recorded the experiment at 25 Hz. This experiment was part of a larger study, parts of which have already been published in a previous publication (Nguyen, Reisner, et al., 2023).

### Measures

#### Maternal singing

Maternal singing was recorded using a microphone (Mangold International), which was placed on a table in the testing room approximately 1 m away from the mother and recorded at a 44,100 Hz sampling rate and pre-processed in Audacity. Excerpts containing audio clipping, infant vocalizations, vegetative noises, and other environmental noises were manually removed. We calculated the following acoustic characteristics of maternal singing per singing condition: spectral flux, amplitude, and pitch using a custom Python script (Python Software Foundation, 2023) and tempo using MIRtoolbox (Lartillot et al., 2008) in Matlab. We opted for tempo extraction via bpm in the MIRtoolbox over Python because of greater accuracy via manual checks. Amplitude was calculated via the amplitude envelope with the numpy package (Harris et al., 2020), pitch via F0 extraction with the parselmouth package (Jadoul et al., 2018). Spectral flux calculations are explained in the Spectrul flux section. We calculated mean spectral flux, amplitude, and pitch as a manipulation check. The main analysis comprised fine-grained acoustic changes in spectral flux relating to infant social gaze onset. As an additional check, we calculated the same fine-grained acoustic changes for amplitude (sound envelope) and pitch (fundamental frequency), which can be found in the Supplemental material (Sections 2.4.S and 2.5.S).

#### Infant gaze

We coded infant gaze as a proxy for infant attention. We distinguished between social gaze (i.e., infant gaze toward the mother’s face) and non-social gaze (i.e., infant gaze oriented away from the mother’s face or body). Only gaze behaviour with a duration of at least 1 s was coded. All videos were coded frame-by-frame and without sound by two principal coders who coded *N* = 30 and *N* = 34 videos, respectively. For assessing inter-rater reliability, two additional independent coders coded *N* = 10 and *N* = 12 videos, respectively, resulting in double coding of 30% of all videos. High inter-rater reliability of a mean of *κ* = .94 was obtained. Descriptive statistics of where infants looked (social vs non-social gaze locations) can be found in Table 2S in the Supplemental material.

#### Spectral flux

Using custom Python code (see data availability below), we extracted spectral flux time series from root-mean-square-normalized audio in maternal singing 5 s before and after infant social gaze onsets. Spectral flux was computed as the sum over frequency (from 0 to 3000 Hz) of the absolute difference in amplitude between consecutive spectra, and in that regard, it measures both overall amplitude and frequency changes over time (Müller, 2015).

$$\mathrm{Spectral}\;\mathrm{Flux}\left(t\right)=\overset{3000Hz}{\underset{f=0Hz}{\sum }}\left|X\left(t;f\right)-\;X(t+0.01;f)\right|$$

*X*(*t; f*) is the spectral amplitude at time *t* and frequency *f* computed over a .03 s window with .02 s overlap, resulting in spectral flux with a temporal resolution of 10 ms. The spectrum was limited to frequencies between 0 and 3000 Hz, as that band contains most of the energy in speech and singing (Lindblom & Sundberg, 2007). We chose spectral flux as an index for acoustic saliency because (1) it measures acoustic variability over time, thus modelling acoustic novelty (Müller & Chiu, 2024), and (2) it has been found to regress better onto brain signals than simple amplitude modulations, as it also measures changes over the frequency domain (Weineck et al., 2022). Especially regarding attention, we think that spectral flux offers a more holistic approach when measuring complex, multi-layered, musical stimuli (Bianco et al., 2025). We excluded instances of gaze onsets where more than 1 s were silent either before or after the gaze onset. This is a methodological consideration to exclude instances in which the spectral flux time series had to be silenced because of other noise sources (environmental sounds, infant vocalizations). Changes in spectral flux were time-locked around gaze onset, synced, and averaged per song type (lullaby or playsong). Surrogate data were simulated by generating 1,000 random onset points per participant and song, excluding time points within 5 s of a social look onset. In the permutation analyses, we compared 10 s windows around infant social gaze onset to surrogate 10 s windows from the same condition not containing infant social gaze. Similar analyses around the infant social gaze offsets can be found in the Supplemental material.

### Statistical analysis

All statistical analysis was done in R Studio (Posit Team, 2024) and Python v3.11.5 (Python Software Foundation, 2023).

We conducted linear or generalized mixed-effects models (LME or GLME, respectively, depending on data distribution) to test for differences in social gaze frequency, absolute duration over the whole song, relative duration (i.e., infant social gaze duration relative to song duration), and individual social look length between the two singing conditions. We calculated the relative duration of infant social gaze because playsongs were significantly longer than lullabies, LME: *χ*^2^(1) = 138.41, *p* < .001, and square-root-transformed the relative duration of looks to aid model convergence. In the Supplemental material (Section 2.2.S), we repeated all analyses with added seat type as a fixed effect to test for differences between infants seated in a car seat vs a highchair.

LME:Socialgazefrequency~songtype+(1|ID)

GLME:Socialgazeabsoluteduration~songtype+(1|ID)

LME:Square-root-transformedsocialgazerelativeduration~songtype+(1|ID)

LME:Log10-transformedindividuallooklengthofsocialgaze~songtype+(1|ID)

We also conducted linear mixed-effects models to look for differences in song length and differences in mean spectral flux, amplitude, pitch, and tempo over the whole playsong and lullaby. In the Supplemental material (Section 2.2.S), we again added seat type as a fixed effect to compare infants seated in a car seat versus a highchair.

LME:Meanspectralflux~songtype+(1|ID)

LME:Meanamplitude~songtype+(1|ID)

LME:Meanpitch,~songtype+(1|ID)

LME:Meantempo~songtype+(1|ID)

Next, we calculated Spearman correlations to test the correlation between infant social gaze frequency, absolute and relative duration, and mean spectral flux in playsongs and lullabies.

We conducted a permutation analysis with independent *t*-tests with custom Python code (see data availability below) to identify time points in the spectral flux 5 s before and after infant social gaze onsets (1,000 permutations, *p*-values below the 5th/2 percentile) in playsongs and lullabies. Similar analyses for amplitude (sound envelope) and pitch (fundamental frequency), as well as for social gaze offsets, can be found in the Supplemental material. For each permutation (*N* = 1,000), a continuous surrogate *p*-value was obtained by randomly re-shuffling the labels of the observed and surrogate look-related changes, and by then performing a series of *t*-tests for the spectral flux over the duration of the look-related changes, resulting in a time series of one truly observed *p*-value and 1,000 re-shuffled *p*-values. We then checked for each time point in the look-related changes whether the truly observed *p*-value was below the Bonferroni-corrected 5th percentile of the re-shuffled *p*-values. We Bonferroni-corrected the 5th percentile for two testing categories (playsong and lullaby). We report significance for time points where the truly observed *p*-value is below the Bonferroni-corrected re-shuffled 5th percentile.

## Results

### Gaze

We compared the frequency, absolute, and relative duration of infant social gaze between playsongs and lullabies to see if infant attention differed between the two conditions (see Table 1). Infant social gaze differed significantly between conditions, with infants showing significantly more instances, *χ*^2^(1) = 12.80, *p* < .001, longer absolute durations, *χ*^2^(1) = 169.98, *p* < .001, longer relative durations, *χ*^2^(1) = 3.94, *p* = .04, and longer individual looks, *χ*^2^(1) = 11.27, *p* < .001, of social gaze toward the mother during playsongs than during lullabies.

**Table 1.** Descriptive statistics on infant social gaze during playsongs and lullabies and acoustic qualities of maternal playsongs and lullabies. Absolute gaze frequency, relative and absolute gaze duration refer to means over the whole condition, duration of individual looks refers to the descriptive statistics of single looking events. Significant condition differences are highlighted in bold (*N* = 74).

	Playsong				Lullaby			
	*M*	*SD*	min	max	*M*	*SD*	min	max
	Infant social gaze							
Absolute frequency	**6.93**	4.00	1.00	17.00	**5.50**	3.25	1.00	17.00
Relative duration (%)	**15.85**	14.63	0.01	68.36	**12.71**	11.69	0.01	71.38
Absolute duration (s)	**27.55**	27.37	1.20	129.00	**17.27**	15.66	1.08	89.00
Duration of individual looks (s)	**4.00**	5.37	1.00	73.36	**3.21**	3.52	1.00	36.34
	Maternal singing							
Song duration (s)	**183.89**	25.11	134.00	238.00	**146.91**	24.94	96.00	207.00
Mean spectral flux (a.u.)	**395.97**	109.81	137.41	672.91	**205.09**	103.71	38.24	668.55
Mean amplitude (a.u.)	**138.84**	28.10	40.91	187.33	**108.61**	29.54	26.34	231.51
Mean pitch (Hz)	249.86	35.44	154.34	337.02	249.77	39.60	153.88	430.14
Mean tempo (bpm)	**162.82**	22.03	109.95	199.65	**136.13**	25.38	95.98	199.63

### Maternal singing

We tested differences in the acoustic qualities of maternal singing over the whole song (see Table 1) to check for mean acoustic differences between the two singing conditions as a manipulation check. Overall, playsongs were significantly longer than lullabies, *χ*^2^(1) = 138.41, *p* < .001, and contained significantly higher mean spectral flux, *χ*^2^(1) = 118.17, *p* < .001, higher mean amplitude, *χ*^2^(1) = 40.69, *p* < .001, and higher tempo, *χ*^2^(1) = 54.76, *p* < .001, than lullabies. There was no significant difference between songs in mean pitch, *χ*^2^(1) = .001, *p* = .981.

Next, we examined the association between spectral flux and infant social gaze. Mean spectral flux positively correlated with absolute duration of infant social gaze (*S* = 400,441, *p* = .033, *ρ* = .178). There was a trend for mean spectral flux to positively correlate with the frequency of infant social gaze (*S* = 412,579, *p* = .067, *ρ* = .153), and with its relative duration (*S* = 418,996, *p* = .095, *ρ* = .14).

### Spectral flux dynamics around infant social gaze

To detect fine time scale acoustic changes in maternal singing and examine whether maternal singing increases in spectral flux around times of increased infant attention, we tested differences between original and surrogate maternal spectral flux 5 s before and after infant social gaze onset.

**Figure 2.**
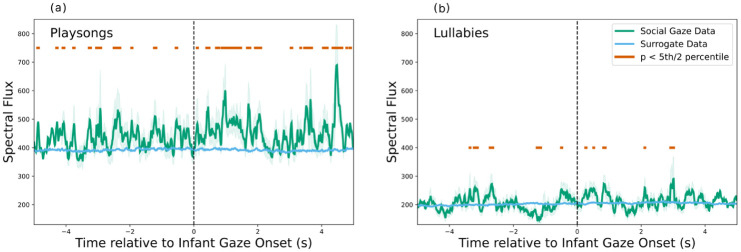
Mean spectral flux playsongs (a) and lullabies (b) 5 s before and after infant social gaze onset (dashed line), depicting instances of infant social gaze toward the mother (green) vs surrogate time points, which excluded time points within 5 s of an infant social gaze onset (blue). Horizontal orange lines indicate time points where the spectral flux around infant social gaze was significantly above or below the spectral flux of non-social surrogate looks.

In playsongs, spectral flux was significantly higher than chance level (*p* < 5th/2 percentile) at time points from T −5.0 to T −2.0 s before, from T −1.0 s before to T + 2.0 s after, and from T + 3.0 to T + 5.0 s after infant social gaze onset (see Figure 2[a] and Table 4S in the Supplemental material). In lullabies, spectral flux was significantly lower than chance level (*p* < 5th/2 percentile) at T −1.5 and T −1.0 s before infant social gaze onset, and significantly above-chance level (*p* < 5th/2 percentile) from T −3.5 to T −2.5 and T −0.5 s before, and at T + 0.5, T + 1.0, T + 2.0, and T + 3.0 s after infant social gaze onset (see Figure 2[b] and Table 4S in the Supplemental material). Similar analyses regarding amplitude (sound envelope) and pitch (fundamental frequency) can be found in the Supplemental material.

## Discussion

This study investigated how mothers and their 7-month-old infants adjusted to each other while the mothers sang a playsong and a lullaby. We aimed to determine whether variability in maternal singing (i.e., spectral flux) changes to modulate or respond to infant attention (i.e., social gaze). Our study revealed two main results. First, infants showed more gaze toward their mothers during playsongs than during lullabies; correspondingly, playsongs contained higher spectral flux than lullabies. Second, mothers showed changes in spectral flux before and after the onsets of infant social gaze in both types of songs. Interestingly, mothers’ peaks in spectral flux as a response to infant attention were especially pronounced in playsongs. These results suggest a reciprocal relationship between mother and infant behaviour in a singing interaction.

Our finding that maternal spectral flux increased before the onset of infant social gaze, especially during playsongs, corroborates previous research showing that caregivers adjust their ID speech by producing rising pitch contours to gain infant eye contact (Fernald et al., 1989; Stern et al., 1982). Thus, mothers may have tried to gain their infants’ attention by increasing the variability in their singing during playsongs more so than during lullabies. This interpretation is further supported by our findings that infants looked longer and more frequently toward their mothers during playsongs than lullabies. Moreover, higher spectral flux across conditions correlated with higher absolute looking duration and marginally correlated with higher relative looking duration and frequency. Indeed, playsongs were found to have higher spectral flux than lullabies, as indicated by more acoustic variability and changes over time. This increased acoustic variability induces auditory uncertainty, which in turn might attract infant attention (Kidd et al., 2014; Poli et al., 2020). Therefore, the more variable playsong was arguably more captivating for the infants, both gaining and maintaining their attention. On the other hand, lullabies could have been acoustically more predictable to fulfil their soothing function (Nguyen, Reisner, et al., 2023; Schwartz, 2004) and thus did not elicit as much attention from the infants. This interpretation is further supported by previous research finding that infants focus their attention more inward during lullabies than during playsongs (Rock et al., 1999). Perhaps even below-baseline arousal responses could further play into this pattern (Cirelli et al., 2020).

Maternal acoustic responses after the onset of infant attention also depended on song type. In playsongs, mothers reciprocated infant attention by increasing spectral flux almost continuously from around 0 to 2 s and 3 to 5 s after infant social gaze onset, possibly to maintain infant attention. Presumably, infant social attention motivated mothers to keep infants engaged with an entertaining and captivating performance, in line with the presumed function of playsongs (Cirelli et al., 2020). In line with these results, previous research findings indicate that caregivers variably adjust their pitch contours in ID speech to maintain infant attention (Stern et al., 1982) and show more frequent and adaptive ID speech depending on whether they can observe their infant’s reciprocal response (Braarud & Stormark, 2008; Lam & Kitamura, 2012; Nencheva & Lew-Williams, 2022; Snow, 1972). In our study, we also found more reciprocal responses in the playsong condition. However, ID singing might be more constrained regarding variable pitch changes than ID speech is (Menn et al., 2022). This constraint on pitch is shown in our supplementary analyses. Amplitude might have been the greater driving factor for variable modulation. In lullabies, on the other hand, mothers did not alter their variability as much as during playsongs after infant social gaze onset, showing very few peaks in spectral flux and even dips (i.e., less variability than in the surrogate data). Mothers might have performed lullabies with lower variability to conform to the song’s intended soothing function. This pattern of results is aligned with a study examining sleeping newborns, showing that caregivers reduce their spectral flux during ID speech to reduce stimulation and increase spectral flux to animate and interact with their newborn infants (Saliba et al., 2020). Further studies could probe into this functionality of ID singing in a more systematic way.

Findings from Bainbridge et al. (2020) suggest that infants show functional differences in their responses even to pre-recorded songs, indicating that reciprocity may not be strictly necessary for some aspects of song function. However, social interaction may serve as an additional driving force in shaping these responses. Specifically, live interactions introduce a feedback loop in which mothers dynamically adapt their singing based on infant cues, amplifying the salience of these responses through social contingency. Prior research on audience effects has demonstrated that live interactions often elicit stronger and more engaged responses compared to recorded stimuli, emphasizing the importance of social presence in communication (Kragness et al., 2023; Rochat, 2001). In this framework, infants are not merely passive recipients of maternal singing but active participants in a reciprocal exchange (Beebe et al., 2016). Furthermore, the extent to which reciprocity influences song function may depend on the type of song—different song types could elicit distinct patterns of adaptation from both mother and child. Thus, while reciprocity may not be an absolute requirement for song function to be realized, it likely enhances its communicative and regulatory effects, reinforcing the interactive nature of caregiver–infant singing.

## Limitations

This study was not without limitations. First, the setup with the tablet was not ideal. While it made it easier to keep infants calm, it could have been distracting to the interaction, and mothers might have had to compete with the tablet for their infants’ attention. However, infants were also looking at the surrounding environment for about a third of the song duration (see Table 2S in the Supplemental material). The tablet was a methodological consideration to reduce infant motion, as we collected infant EEG data in a subset of infants, the results of which can be found in a previous publication (Nguyen, Reisner, et al., 2023). To keep the study design consistent, we opted to keep the tablet in all test sessions. For future studies, we would discourage the use of a tablet if compatible with infant compliance.

Second, technical limitations did not allow us to delve into acoustic variability around beats. We needed a sliding window for our analysis, which was not possible with the MIR toolbox beat extraction, which we chose over Python for greater accuracy. Recent work has shown that the beats of ID singing play an important role both in caregiver behavior (via highlighting salient information) and in infant attentional responses (Lense et al., 2022). Investigating maternal acoustic variability around beats would be an interesting future direction, but we advise to manually code the beats of live singing to ensure maximum accuracy.

Third, this article does not explore the multimodality of maternal signals, such as gestures, facial expressions, or touch. Previous research has shown the importance of eye movements and facial expressions in ID singing (Español & Shifres, 2015; Lense et al., 2022). Future research could expand our study on the interactive nature of ID singing by combining acoustic measures with infant and caregiver behavioral measures to get more insight into the multimodality and possible reciprocity of ID singing.

Fourth, we did not explore trial effects due to not having enough looks per verse. This could be an interesting future direction to uncover where in the song infants paid the most attention.

Furthermore, while mothers sang live to their infants in this study, they were still asked to sing on cue. Especially the lullaby might not have fit the mood of awake infants. Even though there are still significant differences in both maternal acoustic qualities and infant attention, future studies could prioritize even more naturalistic settings, for example, by letting caregivers choose songs that fit their infant’s mood. Recruiting a more diverse sample of caregivers and infants would further enhance the generalizability of results.

## Conclusion

In social interactions, infants are not merely passive observers but are, in fact, actively shaping their interactions (Begus & Southgate, 2018), especially when caregivers reciprocate their social bids (Phillips et al., 2023). This study is the first to show this reciprocity between infants and caregivers in a singing context. Infants not only responded to maternal singing by paying more attention to their variable singing, but mothers also adjusted acoustically to their infants’ social gaze by changing the variability of their singing. This was particularly the case during playsongs. These findings substantiate the distinct functions of ID songs—exciting and attention-grabbing playsongs and calming lullabies. Cross-modal reciprocity between caregivers’ and infants’ social behaviours supports engaging infants and might amplify song function, thus contributing to infant communicative development.

## Supplemental Material

sj-docx-1-msx-10.1177_10298649251385676 – Supplemental material for The reciprocal relationship between maternal infant-directed singing and infant gaze Supplemental material, sj-docx-1-msx-10.1177_10298649251385676 for The reciprocal relationship between maternal infant-directed singing and infant gaze by Susanne Reisner, Trinh Nguyen, Pierre Labendzki, Stefanie Hoehl and Gabriela Markova in Musicae Scientiae

sj-svg-2-msx-10.1177_10298649251385676 – Supplemental material for The reciprocal relationship between maternal infant-directed singing and infant gaze Supplemental material, sj-svg-2-msx-10.1177_10298649251385676 for The reciprocal relationship between maternal infant-directed singing and infant gaze by Susanne Reisner, Trinh Nguyen, Pierre Labendzki, Stefanie Hoehl and Gabriela Markova in Musicae Scientiae

sj-svg-3-msx-10.1177_10298649251385676 – Supplemental material for The reciprocal relationship between maternal infant-directed singing and infant gaze Supplemental material, sj-svg-3-msx-10.1177_10298649251385676 for The reciprocal relationship between maternal infant-directed singing and infant gaze by Susanne Reisner, Trinh Nguyen, Pierre Labendzki, Stefanie Hoehl and Gabriela Markova in Musicae Scientiae

sj-svg-4-msx-10.1177_10298649251385676 – Supplemental material for The reciprocal relationship between maternal infant-directed singing and infant gaze Supplemental material, sj-svg-4-msx-10.1177_10298649251385676 for The reciprocal relationship between maternal infant-directed singing and infant gaze by Susanne Reisner, Trinh Nguyen, Pierre Labendzki, Stefanie Hoehl and Gabriela Markova in Musicae Scientiae

sj-svg-5-msx-10.1177_10298649251385676 – Supplemental material for The reciprocal relationship between maternal infant-directed singing and infant gaze Supplemental material, sj-svg-5-msx-10.1177_10298649251385676 for The reciprocal relationship between maternal infant-directed singing and infant gaze by Susanne Reisner, Trinh Nguyen, Pierre Labendzki, Stefanie Hoehl and Gabriela Markova in Musicae Scientiae

sj-svg-6-msx-10.1177_10298649251385676 – Supplemental material for The reciprocal relationship between maternal infant-directed singing and infant gaze Supplemental material, sj-svg-6-msx-10.1177_10298649251385676 for The reciprocal relationship between maternal infant-directed singing and infant gaze by Susanne Reisner, Trinh Nguyen, Pierre Labendzki, Stefanie Hoehl and Gabriela Markova in Musicae Scientiae

sj-svg-7-msx-10.1177_10298649251385676 – Supplemental material for The reciprocal relationship between maternal infant-directed singing and infant gaze Supplemental material, sj-svg-7-msx-10.1177_10298649251385676 for The reciprocal relationship between maternal infant-directed singing and infant gaze by Susanne Reisner, Trinh Nguyen, Pierre Labendzki, Stefanie Hoehl and Gabriela Markova in Musicae Scientiae

sj-svg-8-msx-10.1177_10298649251385676 – Supplemental material for The reciprocal relationship between maternal infant-directed singing and infant gaze Supplemental material, sj-svg-8-msx-10.1177_10298649251385676 for The reciprocal relationship between maternal infant-directed singing and infant gaze by Susanne Reisner, Trinh Nguyen, Pierre Labendzki, Stefanie Hoehl and Gabriela Markova in Musicae Scientiae

sj-svg-9-msx-10.1177_10298649251385676 – Supplemental material for The reciprocal relationship between maternal infant-directed singing and infant gaze Supplemental material, sj-svg-9-msx-10.1177_10298649251385676 for The reciprocal relationship between maternal infant-directed singing and infant gaze by Susanne Reisner, Trinh Nguyen, Pierre Labendzki, Stefanie Hoehl and Gabriela Markova in Musicae Scientiae
