# Supplementary material for: The reciprocal relationship between maternal infant-directed singing and infant gaze
Source: Music Sci. 2025 Dec 16;30(2):201–14. doi: 10.1177/10298649251385676 (PMC7619065; doi:10.1177/10298649251385676)
Supplement: sj-docx-1-msx-10.1177_10298649251385676 – Supplemental material for The reciprocal relationship between maternal infant-directed singing and infant gaze [file sj-docx-1-msx-10.1177_10298649251385676.docx]

**SUPPLEMENTARY MATERIAL**

1. **S. Methods**
   1. **S. Infant-directed songs**

We selected the German lullaby “Schlaf, Kindlein, schlaf” and the playsong “Es tanzt ein Bibabutzemann”. Each verse was repeated eight times with lyrical variations, resulting in average audio lengths of 148.19 s for the lullaby (range = 96-207 s) and 183.92 s for the playsong (range = 134-238 s).


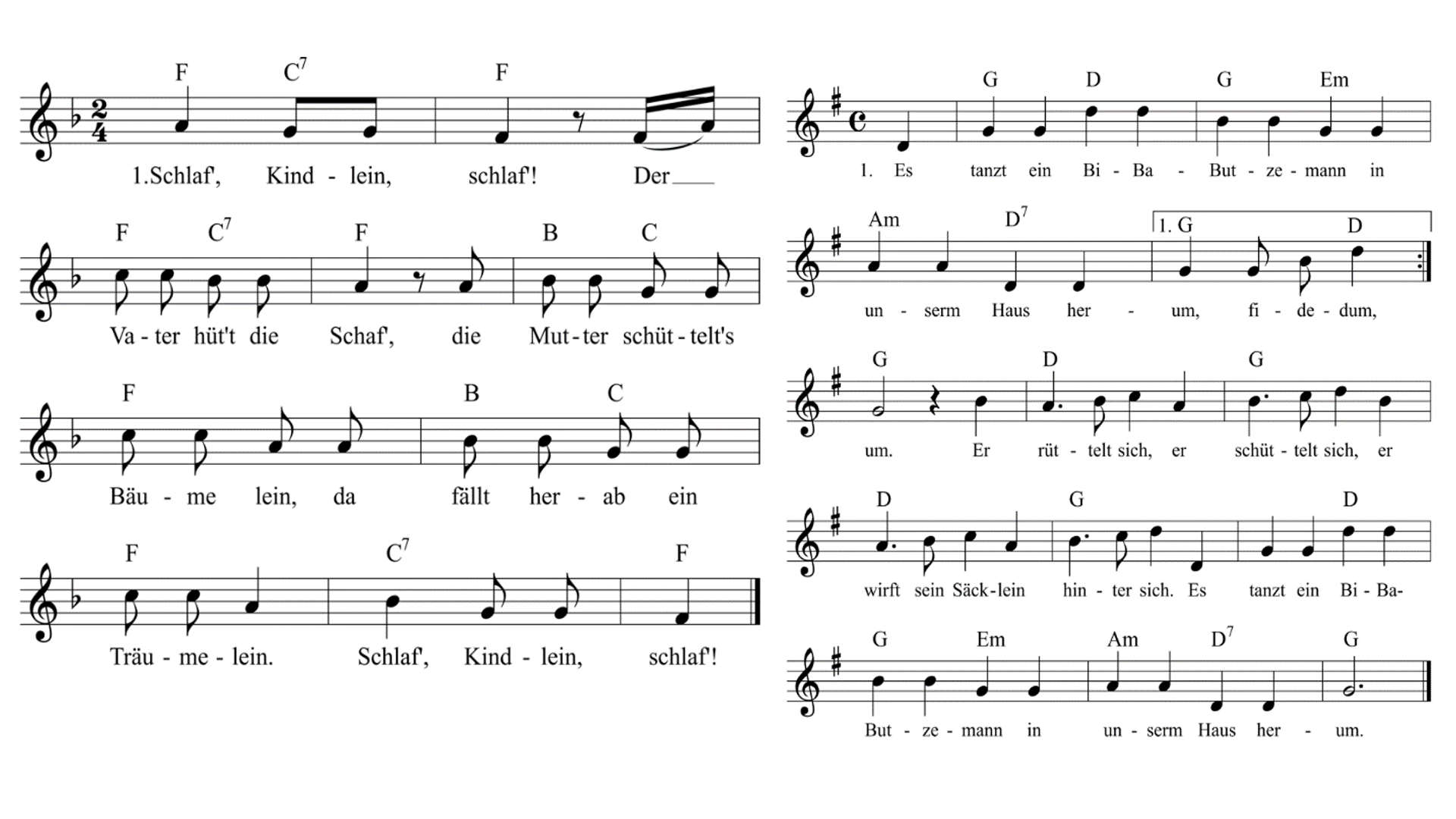


*Figure 1S*. Notations and lyrics of the lullaby (“Schlaf, Kindlein, schlaf”) and the playsong (“Es tanzt ein Bibabutzemann”). Note that mothers were not asked to follow the notations exactly.

- 1. **S. Audio Preparation**

We manually removed infant vocalisations, vegetative noises (e.g., coughs and burps), background noise (i.e., infants hitting against the highchair, metronome ticks), and audio clipping from the audio files using the software Audacity (https://www.audacityteam.org/). To remove audio clipping, i.e., distorted audio due to audio input of too high amplitude, we first detected audio signals of intensities above the 0.95 percentile with a custom Matlab script. We then manually checked these sections for distorted waveforms, which we silenced. For descriptive statistics on the silenced sections, see Table 1S. Two independent observers coded manually removed audio excerpts in 30% of all audios on whether they contained infant vocalisations, yielding high inter-rater reliability with κ = 0.93. The removed contaminations did not differ significantly between the lullaby and playsong conditions, both in frequency (*V* = 1055, *p* = .147) and relative duration (*V* = 1323, *p* = .470).

*Table 1S*. Descriptive statistics on silenced sections in maternal playsongs and lullabies (*N* = 74).

|  | Silenced Sections | | | | | | | |
| --- | --- | --- | --- | --- | --- | --- | --- | --- |
|  | Playsong | | | | Lullaby | | | |
|  | M | SD | min | max | M | SD | min | max |
| Frequency | 20.12 | 17.16 | 0 | 89 | 17.3 | 14.55 | 0 | 65 |
| Relative Duration (%) | 9.1 | 9.1 | 0 | 38.1 | 8.7 | 8 | 0 | 35 |
| Absolute Duration (s) | 16.46 | 16.57 | 0 | 71.6 | 12.89 | 12.59 | 0 | 60.07 |

- 1. **S. Spectral Flux**

*
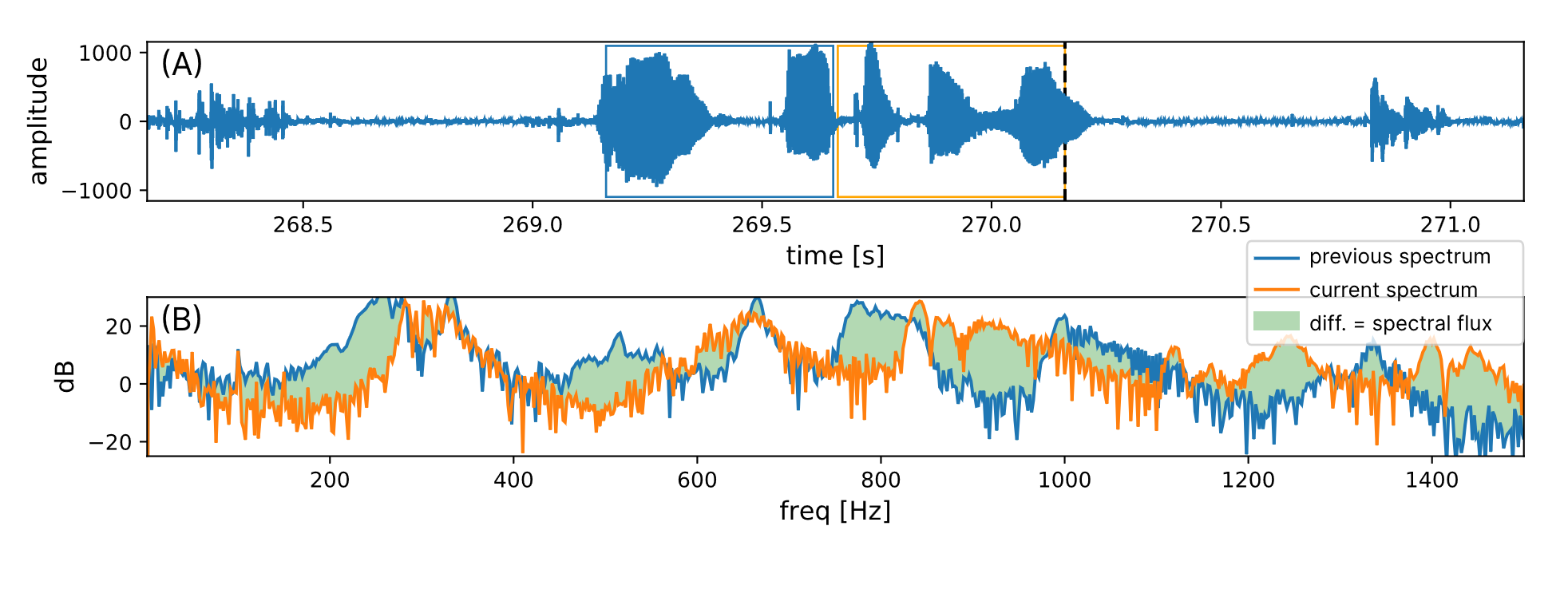
Figure 2*S. Illustration of spectral flux. (A) shows the acoustic waveform over time, with the orange and blue windows indicating respectively the current time window and the previous time window. (B) Current and previous spectra are computed during the time window from (A) using an FFT. Spectral flux is visualised as the green area between the two curves and is formally computed as the frequency-wise difference between consecutive spectra.

1. **S. Results**
   1. **S. Infant Gaze**

*
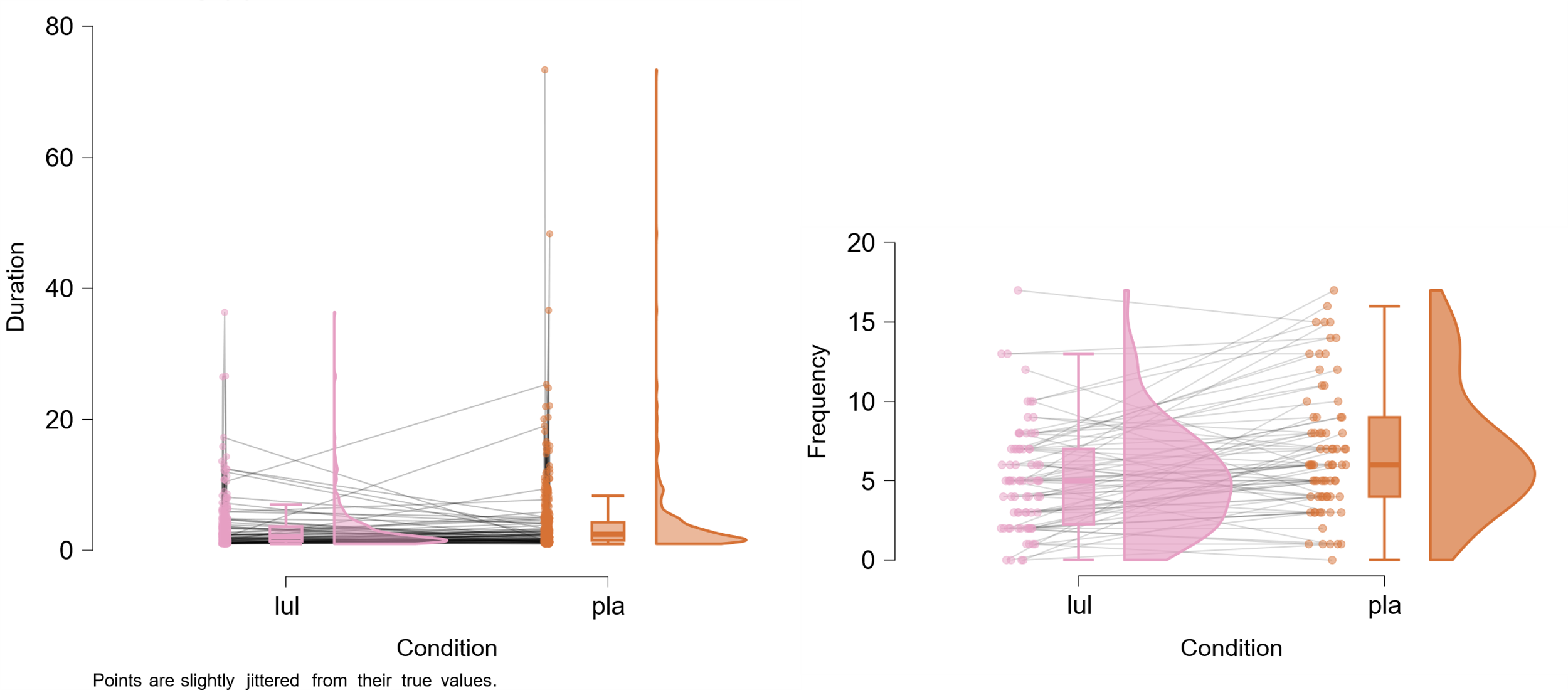
Figure 3S.* Infant social gaze durations and frequency in the two singing conditions (*N* = 74).

*Table 2S*. Descriptive statistics on infant proportional looking duration. Looking behaviour was distinguished between social gaze (towards the mother’s face), non-social gaze (towards the mother’s body, the tablet, away from the mother’s face or body), and not codable (*N* = 74).

|  | Infant Gaze | | | | | | | |
| --- | --- | --- | --- | --- | --- | --- | --- | --- |
|  | Playsong | | | | Lullaby | | | |
|  | M | SD | min | max | M | SD | min | max |
| Mother’s face (%) | 14.9 | 14.4 | 0.0 | 69.1 | 11.7 | 12.4 | 0.0 | 70.4 |
| Mother’s body (%) | 0.1 | 0.5 | 0.0 | 3.4 | 0.1 | 0.3 | 0.5 | 2.2 |
| Tablet (%) | 56.2 | 20.8 | 12.3 | 97.4 | 59.9 | 19.9 | 16.2 | 97.9 |
| Away (%) | 28.1 | 16.9 | 0.0 | 64.4 | 27.5 | 18.3 | 0.0 | 70.1 |
| Not Codable (%) | 0.2 | 1.3 | 0.0 | 10.8 | 0.3 | 1.9 | 0.0 | 16.0 |

Latencies between individual occurrences of infant social looks were variable (see Table 3S). Over both conditions, 187 looks occurred within 5 seconds before the next look onset, and 550 looks occurred after 5 seconds before the next infant social gaze onset.

*Table 3S*. Descriptive statistics on latencies (in seconds) between individual infant social looks (*N* = 74).

|  | Infant Gaze | | | | | | | |
| --- | --- | --- | --- | --- | --- | --- | --- | --- |
|  | Playsong | | | | Lullaby | | | |
|  | M | SD | min | max | M | SD | min | max |
| Latency (s) | 20.48 | 50.31 | 0.76 | 700.96 | 21.71 | 36.01 | 1.1 | 455.72 |

- 1. **S. Effects of Seat Type**

To test the effect of seat type (car seat vs highchair), we added seat type as a fixed effect to our models:

*LME: Social gaze frequency ~ song type + seat type + (1|ID)*

*GLME: Social gaze absolute duration ~ song type + seat type + (1|ID)*

*LME: Square-root-transformed social gaze relative duration ~ song type + seat type*

*+ (1|ID)*

*LME: Log10-transformed individual look length of social gaze ~ song type + seat type*

*+ (1|ID)*

*LME: Mean spectral flux ~ song type + seat type + (1|ID)*

*LME: Mean amplitude ~ song type + seat type + (1|ID)*

*LME: Mean pitch, ~ song type + seat type + (1|ID)*

*LME: Mean tempo ~ song type + seat type + (1|ID)*

Seat type (car seat vs highchair) did not have a significant effect on infant gaze frequency (*χ^2^*(1) = .743, *p* = .389), absolute duration (*χ^2^* (1) = .691, *p* = .406), relative duration (*χ^2^* (1) = 2.63, *p* = .105), or duration of individual social looks (*χ^2^* (1) = 2.736 , *p* = .1). Seat type also did not have a significant effect on song length (*χ^2^* (1) = . 443, *p* = .506), mean spectral flux (*χ^2^* (1) = .677, *p* = .411), mean amplitude (*χ^2^* (1) = .022, *p* = .882), mean tempo (*χ^2^* (1) = 1.242, *p* = .265), or mean pitch (*χ^2^* (1) = .15, *p* = .7).

- 1. **S. Permutation Analyses - Spectral Flux**
     1. **S. Spectral Flux Dynamics Around Infant Social Gaze Onset**

*Table 4S*. Exact time points of above-chance changes in spectral flux in playsongs and lullabies from five seconds before (T -5.0) to five seconds after (T +5.0) the onset of infant social gaze, chunked into half-second intervals. Time points where the observed spectral flux was significantly below the mean surrogate spectral flux are marked with an asterisk (*). The total length (in seconds) of above- or below-threshold changes of every half-second interval is noted in bold and in curly brackets (*N* = 74).

| Time (s) | Playsongs  **{Length (s)}** | Lullabies  **{Length (s)}** |
| --- | --- | --- |
| T -5.0 | [-4.88 – -4.85] **{0.04}** |  |
| T -4.5 | [-4.29 – -4.26]  **{0.09}** |  |
| T -4.0 | [-4.09 – -4.05], [-3.76 – -3.75]  **{0.02}** |  |
| T -3.5 | [-3.74 – -3.73] [-3.27 – -3.25] **{0.05}** | [-3.35 – -3.34]  **{0.02}** |
| T -3.0 | [-3.24 – -3.22], [-3.04 – -3.03], [-2.99 – -2.98],  [-2.94 – -2.90] **{0.12}** | [-3.22 – -3.19], [-3.17 – -3.11] **{0.11}** |
| T -2.5 | [-2.49 – -2.44],[-2.42 – -2.3], [-2.26] **{0.20}** | [-2.72 – -2.63] **{0.10}** |
| T -2.0 | [-1.94 – -1.92] **{0.03}** |  |
| T -1.5 |  | [-1.25]* **{0.01}*** |
| T -1.0 | [-1-23 – -1.17], [-0.94] **{0.08}** | [-1.24 – -1.20]* , [-1.18 – -1.13]* **{0.11}*** |
| T -0.5 | [-0.55 – 0.52], [-0.42]  **{0.05}** | [-0.49 – -0.47] **{0.03}** |
| T 0 | [0.11 – 0.13] **{0.03}** |  |
| T +0.5 | [0.41 – 0.49], [0.71 – 0.73] **{0.12}** | [0.27 – 0.29], [0.42], [0.52 – 0.53]  **{0.06}** |
| T +1.0 | [0.75 – 0.8], [0.89 – 1.06], [1.08 – 1.24] **{0.41}** | [0.83 – 0.89] **{0.07}** |
| T +1.5 | [1.25 – 1.29], [1.31 – 1.48], [1.68 – 1.74] **{0.30}** |  |
| T +2.0 | [1.75 – 1.77], [1.91], [1.93 – 1.94], [1.99 – 2.11] **{0.19}** | [2.12 – 2.13] **{0.02}** |
| T +3.0 | [2.83], [3.05 – 3.08]  **{0.05}** | [2.93 –3.03] **{0.11}** |
| T +3.5 | [3.35 – 3.36], [3.47 – 3.6], [3.64 – 3.70], [3.73] **{0.24}** |  |
| T +4.0 | [3.76], [4.06 – 4.13], [4.17 – 4.19] **{0.12}** |  |
| T +4.5 | [4.36 – 4.39], [4.41 – 4.65] **{0.29}** |  |
| T +5.0 | [4.79 – 4.80], [4.89 – 4.92] **{0.06}** |  |

- - 1. **S. Spectral Flux Dynamics Around Infant Social Gaze Offset**

In playsongs, spectral flux was significantly higher than chance level (*p* <5th/2 percentile), at T -5.0 seconds before and from T -4.0 seconds before to T +5.0 seconds after infant social gaze offset, with only very short periods of above-chance level spectral flux form T 0 to T + 2.0 seconds after infant social gaze offset (see Fig. 4SA and Tab. 5S). In lullabies, only very short periods of significantly higher or lower than chance level (*p* <5th/2 percentile) spectral flux occurred. Spectral flux was lower than chance level at T -5.0 and T -3.0 seconds before infant social gaze offset, and higher than chance level at T -4.0 and T -2.5 seconds before, and at T +1.0, T +2.5, and T +4.0 seconds after infant social gaze offset (see Fig. 4SB and Tab. 5SB).


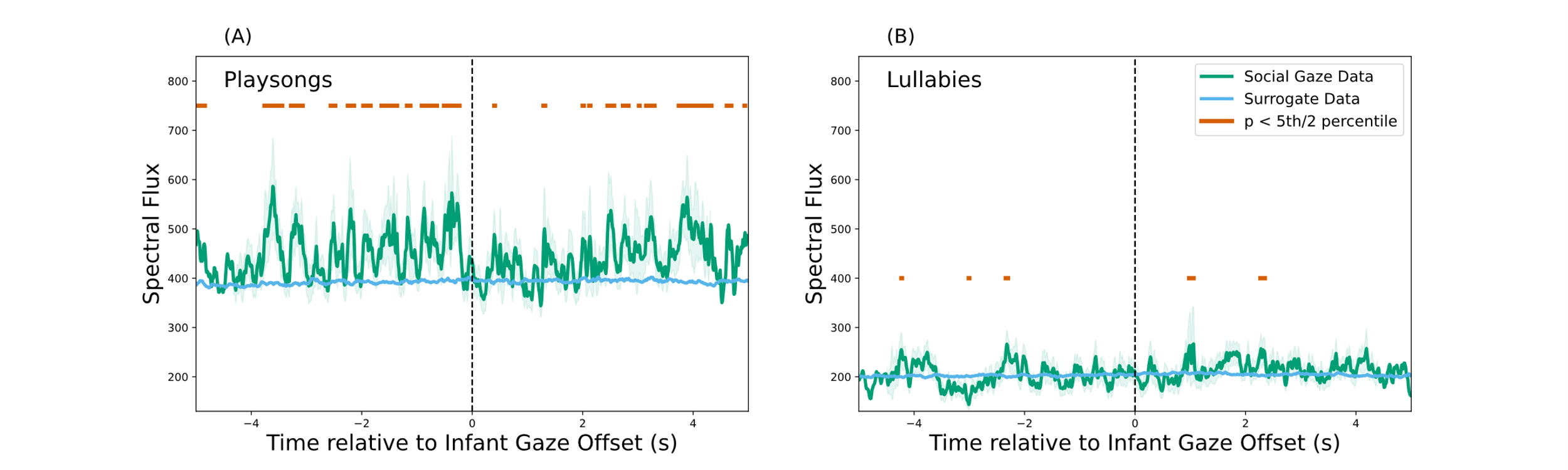


*Figure 4S*. Mean spectral flux playsongs (A) and lullabies (B) 5 s before and after infant social gaze offset (dashed line), depicting instances of infant social gaze towards the mother (green) vs surrogate time points which excluded time points within 5 s of an infant social gaze offset (blue). Horizontal orange lines indicate time points where the spectral flux around infant social gaze was significantly above or below the spectral flux of non-social surrogate looks (*N* = 74).

*Table 5S*. Exact time points of above-chance changes in spectral flux in playsongs and lullabies from five seconds before (T -5.0) to five seconds after (T +5.0) the offset of infant social gaze, chunked into half-second intervals. Time points where the observed spectral flux was significantly below the mean surrogate spectral flux are marked with an asterisk (*). The total length (in seconds) of above- or below-threshold changes of every half-second interval is noted in bold and in curly brackets (*N* = 74).

| Time (s) | Playsongs  **{Length (s)}** | Lullabies  **{Length (s)}** |
| --- | --- | --- |
| T -5.0 | [-4.99 – -4.93], [-4.85 – -4.83] **{0.10}** | [-4.79]* **{0.01}*** |
| T -4.0 | [-3.84], [-3.75]  **{0.02}** | [-4.22 – -4.21], [-3.8] **{0.03}** |
| T -3.5 | [-3.74 – -3.47], [-3.44 – -3.43],  [-3.27 – -3.25] **{0.33}** |  |
| T -3.0 | [-3.24 – -3.06] **{0.19}** | [-3.0 – -2.99]* **{0.02}*** |
| T -2.5 | [-2.55 – -2.47] **{0.09}** | [-2.33 – -2.29] **{0.05}** |
| T -2.0 | [-2.24 – -2.13], [-1.96 – -1.88],  [-1.85 – 1.83] **{0.24}** |  |
| T -1.5 | [-1.63 – -1.49], [-1.47 – -1.39], [-1.36 – -1.35] **{0.26}** |  |
| T -1.0 | [-1.17 – -1.11], [-1.03],  [-0.9 – -0.84], [-0.82 – -0.79] **{0.19}** |  |
| T -0.5 | [-0.74 – -0.63], [-0.5 – -0.39],  [-0.37 – -0.25] **{0.37}** |  |
| T 0 | [-0.24 – -0.22]  **{0.03}** |  |
| T +0.5 | [0.41 – 0.42], [0.52]  **{0.03}** |  |
| T +1.0 | [0.79]  **{0.01}** | [0.99 – 1.01], [1.05 – 1.07]  **{0.06}** |
| T +1.5 | [1.3 – 1.33], [1.38]  **{0.05}** |  |
| T +2.0 | [2.01 – 2.03], [2.05], [2.13 – 2.15]  **{0.07}** |  |
| T +2.5 | [2.46 – 2.58], [2.74]  **{0.14}** | [2.28 – 2.29], [2.32 – 2.33], [2.35 – 2.36]  **{0.06}** |
| T +3.0 | [2.75 – 2.81], [2.83 – 2.84],  [3.03 – 3.04], [3.16 – 3.24]  **{0.20}** |  |
| T +3.5 | [3.25 – 3.31], [3.59]  **{0.08}** |  |
| T +4.0 | [3.75 – 4.21]  **{0.47}** | [4.20]  **{0.01}** |
| T +4.5 | [4.25 – 4.27], [4.32 – 4.34],  [4.62 – 4.66], [4.69 – 4.70]  **{0.13}** |  |
| T +5.0 | [4.94 – 4.95], [4.99]  **{0.03}** |  |

- 1. **S. Permutation analyses - Amplitude (Sound Envelope)**
     1. **S. Amplitude Dynamics Around Infant Social Gaze Onset**

In playsongs, amplitude was significantly higher than chance level (*p* <5th/2 percentile) from T -4.5 to T -3.0, from T -2.0 to T -0.5 seconds before, from T +0.5 to T +2.0, and at T +3.5, T +4.5 and T +5.0 seconds after infant social gaze onset (see Tab. 6S and Fig. 5SA). In lullabies, amplitude was significantly lower than chance level (*p* <5th/2 percentile) at T -1.5 and T -1.0 seconds before infant social gaze onset and significantly higher than chance level (*p* <5th/2 percentile) at T -3.5, T -2.5 seconds before, and at T +0.5, T +1.0, and T +4.5 seconds after infant social gaze onset (see Tab. 6S and Fig. 5SB).

In comparison to spectral flux, there seem to be some overlaps, like above-chance increases in both spectral flux and amplitude envelope around T + 1.0. However, spectral flux seems to have more above-chance changes. This reflects that spectral flux is comprised of more acoustic qualities than just the amplitude envelope.


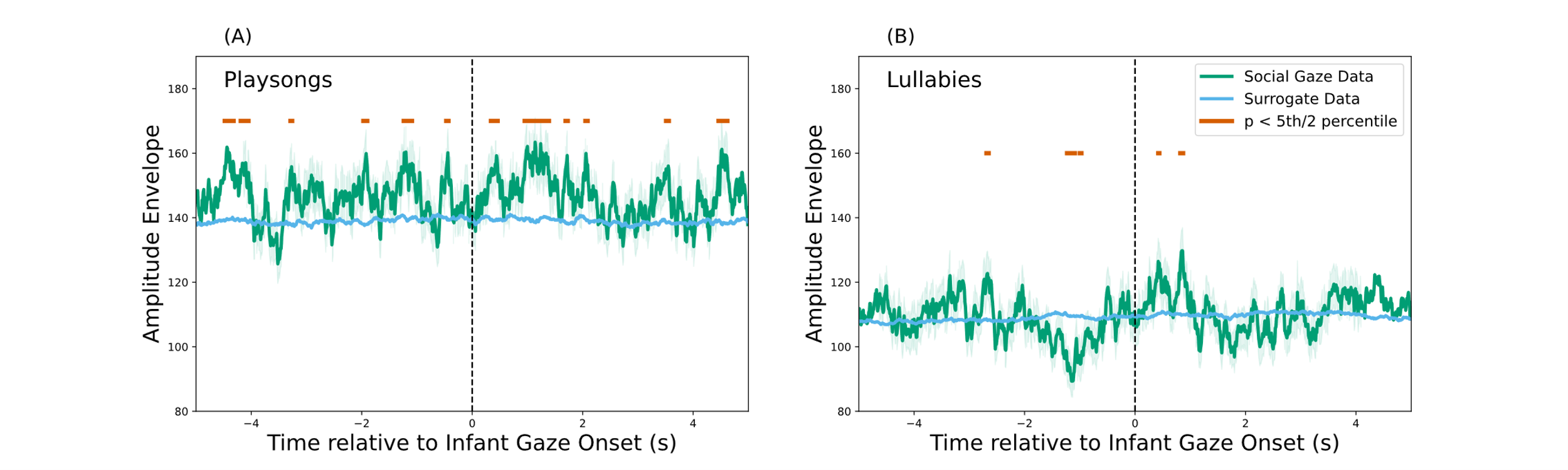


*Figure 5S*. Mean amplitude of playsongs (A) and lullabies (B) five seconds before and after infant social gaze onset (dashed line), depicting instances of infant social gaze towards the mother (green) vs surrogate time points which excluded time points within 5 s of an infant social gaze onset (blue). Horizontal orange lines indicate time points where the amplitude during infant social gaze was significantly above or below the amplitude of surrogate looks (*N* = 74).

*Table 6S*. Exact time points of above-chance changes in amplitude in playsongs and lullabies from five seconds before (T -5.0) to five seconds after (T +5.0) the onset of infant social gaze, chunked into half-second intervals. Time points where the observed spectral flux was significantly below the mean surrogate amplitude are marked with an asterisk (*). The total length (in seconds) of above- or below-threshold changes of every half-second interval is noted in bold and in curly brackets (*N* = 74).

| Time (beats) | Playsongs  **{Length (s)}** | Lullabies  **{Length (s)}** |
| --- | --- | --- |
| T -4.5 | [-4.47 – -4.35],  [-4.32 – -4.31]  **{0.15}** |  |
| T -4.0 | [-4.18 – -4.13],  [-4.11 – -4.09],  [-4.05 – -4.04]  **{0.11}** |  |
| T -3.5 | [-3.28 – -3.25]  **{0.04}** | [-3.34]  **{0.01}** |
| T -3.0 | [-3.21], [-2.90]  **{0.02}** |  |
| T -2.5 |  | [-2.68 – -2.64]  **{0.05}** |
| T -2.0 | [-1.96 – -1.89]  **{0.08}** |  |
| T -1.5 | [-1.31], [-1.27], [-1.25]  **{0.03}** | [-1.44]*, [-1.27]*, [-1.25]*  **{0.03}*** |
| T -1.0 | [-1.23 – -1.15],  [-1.12 – -1.11],  [-1.09 – -1.08], [-1.06],  [-0.94]  **{0.15}** | [-1.22 – -1.08]*, [-1.03]*,  [-0.99 – -0.96]*  **{0.20}*** |
| T -0.5 | [-0.46 – -0.42]  **{0.05}** |  |
| T +0.5 | [0.35 – 0.40], [0.42 – 0.47]  **{0.12}** | [0.43 – 0.45]  **{0.03}** |
| T +1.0 | [0.96 – 1.24]  **{0.28}** | [0.81], [0.83 – 0.88]  **{0.07}** |
| T +1.5 | [1.25 – 1.29], [1.31 – 1.40], [1.70 – 1.74]  **{0.20}** |  |
| T +2.0 | [2.04], [2.06 – 2.10]  **{0.06}** |  |
| T +3.5 | [3.37], [3.52 – 3.57]  **{0.07}** |  |
| T +4.5 | [4.47 – 4.48], [4.50 – 4.63]  **{0.16}** | [4.35]  **{0.01}** |
| T +5.0 | [4.90]  **{0.01}** |  |

- - 1. **S. Amplitude Dynamics Around Infant Social Gaze Offset**

In playsongs, amplitude was significantly higher than chance level (*p* <5th/2 percentile) from T -4.0 seconds to T 0 seconds before, and from T +2.5 to T +4.5 seconds after infant social gaze offset (see Tab. 7S and Fig. 6SA). In lullabies, amplitude was significantly lower than chance level (*p* <5th/2 percentile) at T -3.5, T -3.0, T -0.5 seconds before, and at T +0.5 seconds after the onset of infant social gaze offset, and significantly higher than chance level at T -2.5 seconds before, at T +2.0 to T +3.0, and at T +4.0 seconds after infant social gaze offset. (see Tab. 7S and Fig. 6SB)


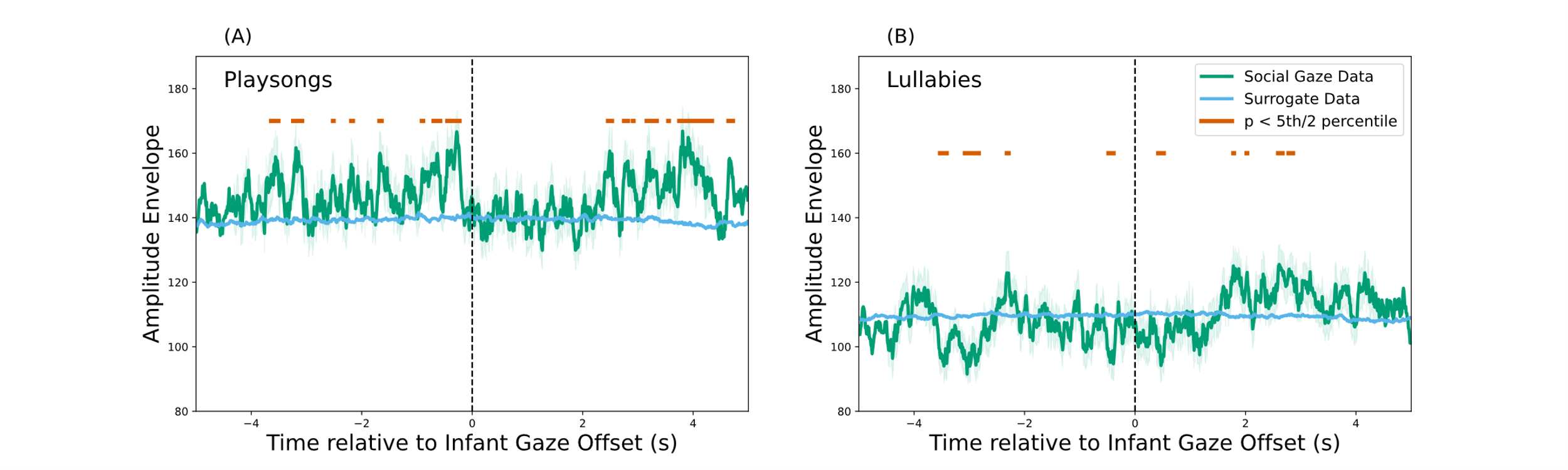


*Figure 6S*. Mean amplitude (sound envelope) in playsongs (A) and lullabies (B) five seconds before and after infant social gaze offset (dashed line), depicting instances of infant social gaze towards the mother (green) vs surrogate time points which excluded time points within 5 s of an infant social gaze offset (blue). Horizontal orange lines indicate time points where the pitch during infant social gaze was significantly above or below the pitch of non-social surrogate looks (*N* = 74).

*Table 7S*. Exact time points of above-chance changes in amplitude in playsongs and lullabies from five seconds before (T -5.0) to five seconds after (T +5.0) the offset of infant social gaze, chunked into half-second intervals. Time points where the observed spectral flux was significantly below the mean surrogate amplitude are marked with an asterisk (*). The total length (in seconds) of above- or below-threshold changes of every half-second interval is noted in bold and in curly brackets (*N* = 74).

| Time (beats) | Playsongs  **{Length (s)}** | Lullabies  **{Length (s)}** |
| --- | --- | --- |
| T -4.0 | [-4.04]  **{0.01}** |  |
| T -3.5 | [-3.72], [-3.70], [-3.66],  [-3.63 – -3.60],  [-3.58 – -3.50], [-3.44]  **{0.17}** | [-3.52 – -3.43]*, [-3.41 – -3.40]*  **{0.12}*** |
| T -3.0 | [-3.23 – -3.07]  **{0.17}** | [-3.09]*, [-3.07 – -3.01]*,  [-2.98 – -2.95]*, [-2.89 – -2.82]*  **{0.20}*** |
| T -2.5 | [-2.51 – -2.50]  **{0.02}** | [-2.31 – -2.28]  **{0.04}** |
| T -2.0 | [-2.22], [-2.18 – -2.15]  **{0.05}** |  |
| T -1.5 | [-1.67 – -1.63], [-1.60],  [-1.53]  **{0.07}** |  |
| T -1.0 | [-0.9 – -0.88]  **{0.03}** |  |
| T -0.5 | [-0.72], [-0.69 – -0.68],  [-0.66 – -0.57],  [-0.44 – -0.34]  [-0.31 – -0.25]  **{0.31}** | [-0.47 – -0.46]*, [-0.44 – -0.41]*,  [-0.39 – -0.38]*  **{0.08}*** |
| T 0 | [-0.24 – -0.22]  **{0.03}** |  |
| T +0.5 |  | [0.43 – 0.44]*, [0.47 – 0.50]*,  [0.52 – 0.53]*  **{0.08}*** |
| T +1.0 |  | [1.18]*  **{0.01}*** |
| T +1.5 |  |  |
| T +2.0 |  | [1.77], [1.79 – 1.80], [1.83], [1.85], [1.89], [2.03 – 2.04]  **{0.08}** |
| T +2.5 | [2.47 – 2.51], [2.53 – 2.54]  **{0.07}** | [2.33], [2.60 – 2.68], [2.70]  **{0.11}** |
| T +3.0 | [2.76 – 2.83], [2.87],  [2.92 – 2.93], [3.17 – 3.22]  **{0.17}** | [2.79 – 2.87], [3.18]  **{0.10}** |
| T +3.5 | [3.25 – 3.26], [3.28 – 3.35],  [3.51], [3.56 – 3.57], [3.64],  [3.66]  **{0.15}** |  |
| T +4.0 | [3.76 – 4.05], [4.07 – 4.19], [4.21 – 4.22]  **{0.45}** | [4.17], [4.23]  **{0.02}** |
| T +4.5 | [4.27 – 4.28], [4.33 – 4.35], [4.65 – 4.73]  **{0.14}** |  |

- 1. **S. Permutation analyses – Pitch (Fundamental Frequency)**
     1. **S. Pitch Dynamics Around Infant Social Gaze Onset**

In playsongs, pitch was significantly lower than chance level (*p* <5th/2 percentile) at T -1.5 seconds before and T +4.0 seconds after infant social gaze onset (see Tab. 8S and Fig. 7SA). In lullabies, amplitude was significantly lower than chance level (*p* <5th/2 percentile) at T -4.5 and T -0.5 seconds before infant social gaze onset (see Tab. 8S and Fig. 7SB).

The sporadicity of robust pitch changes could be due to songs having a distinct pitch that mothers could not vary as much as amplitude or spectral flux in response to infant attention.


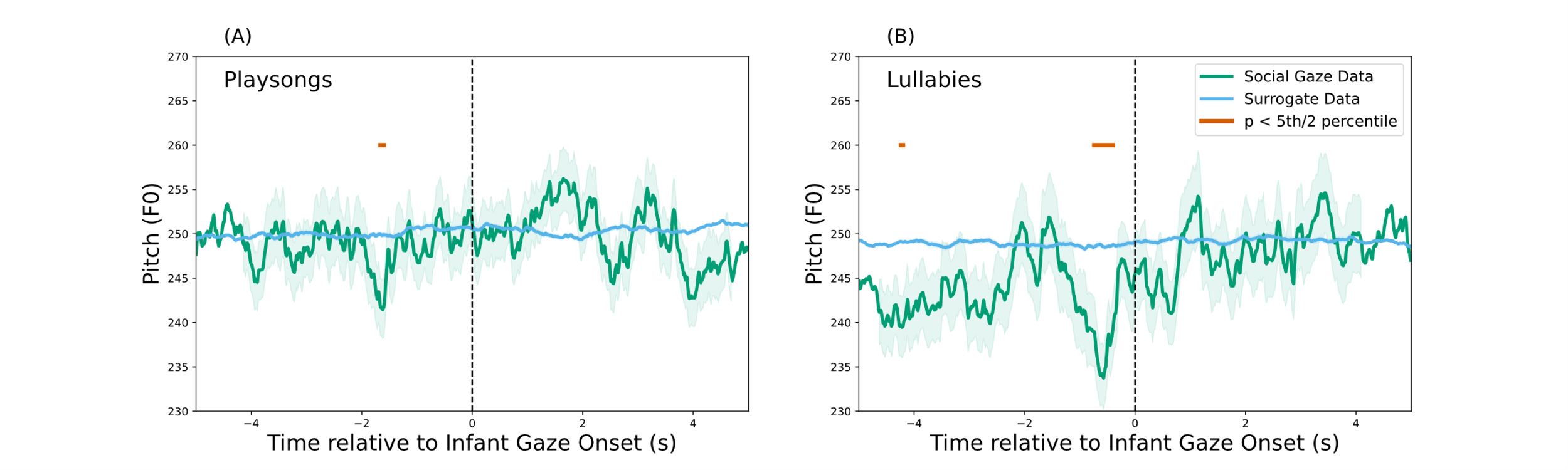


*Figure 7S*. Mean pitch (fundamental frequency) in playsongs (A) and lullabies (B) five seconds before and after infant social gaze onset (dashed line), depicting instances of infant social gaze towards the mother (green) vs surrogate time points which excluded time points within 5 s of an infant social gaze onset (blue). Horizontal orange lines indicate time points where the pitch during infant social gaze was significantly below the pitch of non-social surrogate looks (*N* = 74).

*Table 8S*: Exact time points of above-chance changes in pitch in playsongs and lullabies from five seconds before (T -5.0) to five seconds after (T +5.0) the onset of infant social gaze, chunked into half-second intervals. Time points where the observed spectral flux was significantly below the mean surrogate pitch are marked with an asterisk. The total length in seconds of every half-second interval is noted in bold and in curly brackets (*N* = 74).

| Time (beats) | Playsongs  **{Length (s)}** | Lullabies  **{Length (s)}** |
| --- | --- | --- |
| T -4.0 |  | [-4.23 – -4.19]*  **{0.05}*** |
| T -1.5 | [-1.65 – -1.59]*  **{0.07}*** |  |
| T -0.5 |  | [-0.73 – -0.39]*  **{0.35}*** |

- - 1. **S. Pitch Dynamics Around Infant Social Gaze Offset**

In playsongs, pitch was not significantly different from chance level (*p* <5th/2 percentile) around infant social gaze offset (see Tab. 9S and Fig. 8SA). In lullabies, pitch was significantly lower than chance level (*p* <5th/2 percentile) at T -5.0 before the offset of infant social gaze (see Tab. 9S and Fig. 8SB).


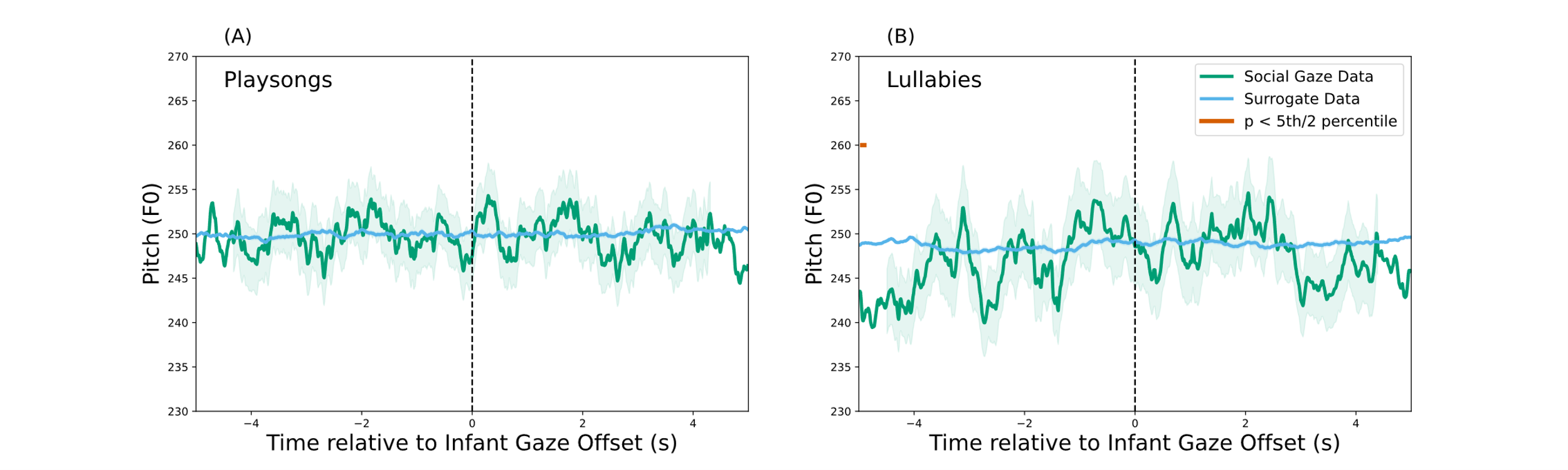


*Figure 8S*. Mean pitch (fundamental frequency) in playsongs (A) and lullabies (B) five seconds before and after infant social gaze offset (dashed line), depicting instances of infant social gaze towards the mother (green) vs surrogate time points which excluded time points within 5 s of an infant social gaze offset (blue). Horizontal orange lines indicate time points where the pitch during infant social gaze was significantly below the pitch of non-social surrogate looks (*N* = 74).

*Table 9S*. Exact time points of above-chance changes in pitch (fundamental frequency) in playsongs and lullabies from five seconds before (T -5.0) to five seconds after (T +5.0) the offset of infant social gaze, chunked into half-second intervals. Time points where the observed spectral flux was significantly below the mean surrogate pitch are marked with an asterisk. The total length in seconds of every half-second interval is noted in bold and in curly brackets (*N* = 74).

| Time (beats) | Playsongs  **{Length (s)}** | Lullabies  **{Length (s)}** |
| --- | --- | --- |
| T -5.0 |  | [-4.93 – -4.89]*  **{0.05}*** |
